# NLRX1 Enhances Glutamate Uptake and Inhibits Glutamate Release by Astrocytes

**DOI:** 10.3390/cells8050400

**Published:** 2019-04-30

**Authors:** Shaimaa Mahmoud, Marjan Gharagozloo, Camille Simard, Abdelaziz Amrani, Denis Gris

**Affiliations:** 1 Program of Immunology, Department of Pharmacology-Physiology, Faculty of Medicine and Health Sciences, University of Sherbrooke, Sherbrooke, QC J1H 5N4, Canada; Shaimaa.Mahmoud@usherbooke.ca (S.M.); Marjan.Gharagozloo@usherbrooke.ca (M.G.); Camille.Simard@usherbrooke.ca (C.S.); 2 Program of Immunology, Department of Pediatrics, CR-CHUS, Faculty of Medicine and Health Sciences, University of Sherbrooke, Sherbrooke, QC J1H 5N4, Canada; Abdelaziz.Amrani@usherbrooke.ca

**Keywords:** NLRX1, astrocytes, CNS, glutamate uptake, glutamate release, excitotoxicity

## Abstract

Uptake of glutamate from the extracellular space and glutamate release to neurons are two major processes conducted by astrocytes in the central nervous system (CNS) that protect against glutamate excitotoxicity and strengthen neuronal firing, respectively. During inflammatory conditions in the CNS, astrocytes may lose one or both of these functions, resulting in accumulation of the extracellular glutamate, which eventually leads to excitotoxic neuronal death, which in turn worsens the CNS inflammation. NLRX1 is an innate immune NOD-like receptor that inhibits the major inflammatory pathways. It is localized in the mitochondria and was shown to inhibit cell death, enhance ATP production, and dampen oxidative stress. In the current work, using primary murine astrocyte cultures from WT and *Nlrx1^-/-^* mice, we demonstrate that NLRX1 potentiates astrocytic glutamate uptake by enhancing mitochondrial functions and the functional activity of glutamate transporters. Also, we report that NLRX1 inhibits glutamate release from astrocytes by repressing Ca^2+^-mediated glutamate exocytosis. Our study, for the first time, identified NLRX1 as a potential regulator of glutamate homeostasis in the CNS.

## 1. Introduction

Astrocytes are the most numerous glial cell type in the central nervous system (CNS). They perform many pivotal functions associated with neuronal support and maintenance of the CNS homeostasis [1]. One of these crucial functions is to uptake excess synaptically-released glutamate (the major excitatory neurotransmitter in the CNS) [2,3] from the extracellular space, metabolize it, and send it back to neurons [4,5]. This glutamate uptake is mediated primarily by the excitatory amino acid transporters 1 and 2 (EAAT1 and EAAT2), expressed by astrocytes (known in mice as glutamate-aspartate transporter (GLAST) and glutamate transporter-1 (GLT-1), respectively) [6,7,8,9,10,11]. In astrocytes, under physiological conditions, the uptake of glutamate against its concentration gradient relies on glutamate transporters and Na^+^/K^+^ ATPase that consumes high levels of intracellular adenosine triphosphate (ATP) [12,13]. Many factors in the CNS influence the expression, trafficking, and functional activity of glutamate transporters in astrocytes, including hormones, growth factors, inflammatory mediators, and oxidative stress [14,15,16].

Recent studies suggested that, along with glutamate uptake, astrocytes release glutamate, which helps synchronize and intensify firing of the surrounding neurons [4,17,18]. Astrocytic glutamate release is mediated mostly by Ca^2+^-dependent exocytosis [19]. In astrocytes, part of the sequestrated glutamate is transferred into small intracellular vesicles [20,21] by activation of the vesicular glutamate transporters 1 and 2 (VGLUT1 and VGLUT2), derived by the proton gradient generated by the vacuolar (H^+^) ATPase (V-ATPase) [22,23,24]. Astrocytes also express a Ca^2+^ sensor (synaptotagmin 4, 7, or 11) [25,26,27] and vesicular fusion proteins (vesicle-associated membrane protein 2 and 3 (VAMP2 and VAMP3)) [20,28]. In response to intracellular Ca^2+^ elevation, caused by Ca^2+^ release from the endoplasmic reticulum (ER), vesicular fusion proteins (VAMP2 and VAMP3) fuse with the astrocyte cell membrane fusion proteins (syntaxin and soluble N-ethylmaleimide-sensitive factor attachment protein 23 (SNAP23)) [29,30,31], resulting in glutamate release from the vesicles into the extracellular space.

In the context of CNS pathologies, astrocytes respond to inflammation by losing some of their vital functions or acquiring some deleterious effects that aggravate inflammatory conditions in the CNS and delay the processes of recovery [1]. Previous studies showed that neuropathological conditions in the CNS, such as brain trauma, infection by human immunodeficiency virus (HIV), multiples sclerosis (MS), amyotrophic lateral sclerosis (ALS), Alzheimer’s disease (AD), and Parkinson’s disease (PD) are associated with accumulation of glutamate in the extracellular space, caused by reduced glutamate uptake and/or increased glutamate release by astrocytes [32,33,34,35,36,37]. Excess extracellular glutamate induces hyperstimulation of glutamate receptors in neurons and eventually leads to neuronal death, in a process known as “glutamate excitotoxicity” [38]. The excess of extracellular glutamate and subsequent neuronal death, in turn, enhance the inflammatory response and worsen the pathological conditions in the CNS [38].

NOD-like receptors (NLRs) are cytosolic innate immune molecules that can exert either positive or negative effects on inflammation in the CNS [39]. NLRX1 is a recently discovered anti-inflammatory NLR that inhibits nuclear factor-κB (NF-κB) and RIG-1-MAVs signaling pathways, thus inhibiting antimicrobial immune responses [40,41,42,43,44] and sterile inflammations [45]. Since the introduction of *Nlrx1^-/-^* mice, many studies implicated NLRX1 in the development of various pathologies. For example, *Nlrx1^-/-^* mice show excessive inflammatory response following Influenza virus infection and LPS treatment [41]. Also, *Nlrx1^-/-^* mice show exacerbated severity of inflammatory bowel disease (IBD) [46] and increased incidence of colitis-associated colonic cancer [45]. In the CNS, lack of Nlrx1 in mice is associated with an excessive inflammation following CNS trauma [47], earlier onset, and a more aggressive course of the experimental autoimmune encephalomyelitis (EAE), a mouse model of MS [48]. Moreover, using a neuroblastoma cell line, NLRX1 was shown to inhibit neuronal death and redirect rotenone-treated neurons towards apoptosis instead of necrosis [49]. Unlike other NLRs, NLRX1 is located at the mitochondria. It enhances various mitochondrial functions and activities such as ATP production and respiration while inhibiting oxidative stress and apoptosis [44,49,50,51,52,53,54].

In the current study, we investigated the role of NLRX1 in glutamate uptake and release by primary murine astrocytes, and the potential mechanisms by which NLRX1 mediates its effects.

## 2. Materials and Methods

### 2.1. Mice

All mice handling and manipulations were approved by the Institutional Animal Care and Use Committee at the University of Sherbrooke (Protocols #280-15, 4 April 2017) according to the Canadian Council on Animal Care. All mice were bred on C57/BL6J background. Wild-type (WT) mice were bred in-house in the same conditions as *Nlrx1^-/-^* mice that were kindly provided by Dr. Jenny P. Y. Ting (Chapel Hill, NC, USA). 

### 2.2. Primary Mouse Astrocyte Cultures

Glial cultures were prepared from 1-day-old pups, as previously described [55]. Pups were sacrificed by decapitation, and brains were harvested and placed in 100 mm culture plates. Brain tissue was dissociated by a commercial razor blade, followed by triturating in 10 mL DMEM/F12 medium (Wisent Inc., Montreal, QC, Canada) containing 10% deactivated fetal bovine serum (dFBS), 2 mM l-glutamine, 1% MEM amino acid, 1% sodium pyruvate, and 1% penicillin-streptomycin and amphotericin B (all from Wisent Inc., Montreal, QC, Canada). Dissociated tissue was passed through 70 µm cell strainer to remove tissue debris. Cells were plated in 100 mm cell culture plates (Corning Inc., Brooklyn, NY, USA) with DMEM/F12 complete medium and incubated in 37 °C incubator with 5% CO_2_. The medium was changed every 2–3 days to wash out cells other than glial cells. After 21 days, glial cultures were resuspended in 10% dimethyl sulfoxide (DMSO) in dFBS (freezing medium) and were frozen at −80 °C. One week before the experiments, cells were thawed and reseeded in 100 mm culture plates, in complete DMEM/F12 medium. Cells were stained with CD11b (eBioscience/Thermofisher scientific, Waltham, Massachusetts, USA # 12-0112-81) as a marker for microglia and the percentage of CD11b-expressing cells was measured by flow cytometry. In our experiments, we used cultures containing less than 10% CD11b+ cells (astrocytes ≥90%) since additional purification of astrocytes did not affect the glutamate uptake or release.

### 2.3. Glutamate Uptake and Release Assay

The assay was modified from Piao et al. 2015 [56]. 100,000 astrocytes were seeded in each well of a 96-well plate, and washed 2 times with Hank’s Balanced Salt Solution (HBSS) containing Ca^2+^ (Wisent Inc., Montreal, QC, Canada): 1.26 mM CaCl_2_ (anhydrous), 5.36 mM KCl, 0.44 mM KH_2_PO_4_, 0.811 mM MgSO_4_ (anhydrous), 137 mM NaCl, 0.336 mM Na_2_HPO_4_ (anhydrous), 4.166 mM NaHCO_3_, and 5.55 mM d-glucose, pH 7.25 ± 0.15 or Ca^2+^-free Lock’s solution: 140 mM NaCl, 4.7 mM KCl, 1.2 mM KH_2_PO_4_, 1.2 MgSO_4_, 11 mM glucose, and 15 mM HEPES-NaOH. For glutamate uptake, cells were incubated with 100 or 200 µM glutamate in the Ca^2+^-containing HBSS for 4 h, while for glutamate release, astrocytes were incubated in Ca^2+^-containing HBSS or Ca^2+^-free Lock’s solution for 1 h, in the 37 °C with 5% CO_2_ incubator. Then, culture supernatant was collected, and glutamate concentration in the medium was measured using a glutamate colorimetric assay kit (Sigma-Aldrich, Oakville, ON, Canada # MAK004 or Abcam, Toronto, ON, Canada #ab83389) according to the manufacturer’s instructions. Glutamate uptake by astrocytes was measured by subtracting the amount of glutamate measured in the medium from the amount initially added to the cells. Both glutamate uptake and release by astrocytes were normalized to the amount of protein in the corresponding cells, measured by Bradford assay (Bio-Rad, Montreal, QC, Canada).

### 2.4. Quantitative Real-Time PCR (qPCR)

RNA was extracted from astrocytes using TRIzol reagent (Life Technologies Inc./Thermofisher Scientific, Waltham, MA, USA # 15596-018) according to the manufacturer’s instructions. cDNA was synthesized from RNA, using Oligo(dT) primer (IDT, Coralville, IA, USA), dNTP Mix, M-MuLV Reverse Transcriptase, M-MuLV Reverse Transcriptase Buffer, and RNase inhibitor (all from New England Biolabs, Whitby, ON, Canada), as previously described [55]. qPCR was performed using KiCqStart^®^ SYBR^®^ Green qPCR ReadyMix (Sigma-Aldrich, Oakville, ON, Canada # KCQS00). The ΔΔC_T_ method was used to calculate the relative gene expression to *18S* as a housekeeping gene [57]. Primer sequences used (IDT, Coralville, IA, USA) are shown in Table 1. 

### 2.5. Flow Cytometry Staining

To measure the total protein expression of glutamate transporters, intracellular staining was performed (protocol modified from Gharagozloo et al. 2018 and Schwarz et al. 2013) [58,59]. WT and *Nlrx1^-/-^* astrocytes were washed with phosphate-buffered saline (PBS), fixed, permeabilized, and blocked with 5% dFBS in washing buffer. Cells were stained with the anti-GLT-1 antibody (Novus Biologicals, Centennial, CO, USA # NBP1-20136) diluted 1:100 or anti-GLAST antibody (Novus Biologicals, Centennial, CO, USA # NB100-1869) diluted 1:200 and incubated for 30–40 min. Astrocytes were washed twice and incubated with the secondary anti-rabbit IgG antibody, Alexa Fluor^®^ 555 Conjugated (New England Biolabs, Whitby, ON, Canada #4413) diluted 1:1000, for 20 min. Cells were washed twice after the secondary antibody and resuspended in PBS.

To measure the cell surface expression of the transporters, astrocytes were washed and stained using the same previous procedure, but with no cell fixation or permeabilization.

To detect the activity of reactive oxygen species (ROS), dihydrorhodamine 123 (DHR) was added to the cells to a final concentration of 0.5 µg/mL and incubated for 15 min at 37 °C, then cells were resuspended in PBS (protocol modified from Gris et al. 2008 and Farrell et al. 2011) [60,61].

Sample acquisition was realized using Beckman Coulter CytoFlex (Beckman Coulter, Brea, CA, USA). Data analysis was performed, and histograms produced using CytExpert 2.3 software (Beckman Coulter, Brea, CA, USA).

### 2.6. Measurement of Intracellular ATP

The assay was performed using the ATP bioluminescent assay kit (Sigma-Aldrich, Oakville, ON, Canada # FLAA) according to the manufacturer’s instructions, modified from Marcaida et al. 1997 [62]. 100,000 astrocytes from WT and *Nlrx1^-/-^* mice were lysed with 400 µL of somatic cell ATP-releasing reagent (Sigma-Aldrich, Oakville, ON, Canada # FLSAR). In a white opaque 96-well plate, 100 µL of the ATP reaction mix was added to each well and incubated for 3 min. Samples (100 µL) from the ATP standard or the cell lysate were added to the reaction mix and vigorously mixed. Immediately, using a luminometer, the amount of light produced from the reaction was measured, which reflected the amount of ATP in each well.

### 2.7. Measurement of Mitochondrial DNA (mtDNA)

DNA was extracted from astrocytes using TRIzol reagent (Life Technologies Inc./Thermofisher Scientific, Waltham, MA, USA # 15596-018) according to the manufacturer’s instructions. qPCR was performed, as previously described, to compare the relative amount of mtDNA between WT and *Nlrx1^-/-^* astrocytes [63], using 100 ng of the extracted DNA and KiCqStart^®^ SYBR^®^ Green qPCR ReadyMix (Sigma-Aldrich, Oakville, ON, Canada # KCQS00). To estimate the amount of mtDNA, the mitochondrial DNA region (*D-loop*) was amplified, using the two primer sequences: D1 (5′-CCC AAG CAT ATA AGC TAG TAC-3′) and D2 (5′-ATA TAA GTC ATA TTT TGG GAA CTA C-3′), with the thermal cycling protocol 95 °C for 20 s, 55 °C for 20 s, 72 °C for 80 s for 30 cycles after an initial denaturation. To estimate the amount of nuclear DNA as a reference, the (*apo-B*) region was amplified, using the two primer sequences: 5′-CGT GGG CTC CAG CAT TCT A-3′ and 5′-TCA CCA GTC ATT TCT GCC TTT G-3′, with the two-step thermal cycling protocol 95 °C for 10 s and 60 °C for 30 s for 40 cycles after an initial denaturation at 95 °C for 1 min. The relative amount of mtDNA to the nuclear DNA was calculated by the ΔΔC_T_ method.

### 2.8. Statistical Analysis

All statistical analysis was conducted using GraphPad Prism 8 software (GraphPad, San Diego, CA, USA). Results were expressed as the mean ± standard error of the mean (SEM). Statistical differences between WT and *Nlrx1^-/-^* astrocytes were assessed by Mann–Whitney test. Glutamate uptake and glutamate release using different treatments were assessed by two-way ANOVA followed by Tukey’s test. The significance level was set at *p* < 0.05.

## 3. Results

### 3.1. NLRX1 Inhibits Excess Glutamate Release and Enhances Glutamate Uptake by Astrocytes

**Figure 1:**
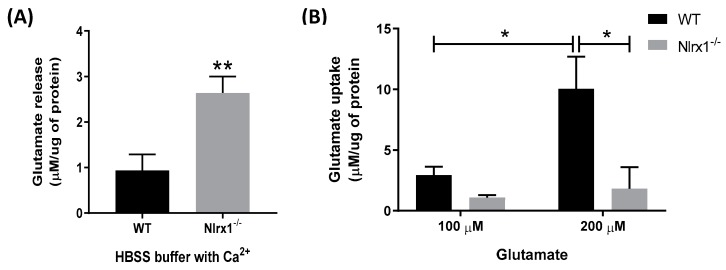
NLRX1 inhibits glutamate release and enhances glutamate uptake by astrocytes. (**A**) WT and *Nlrx1^-/-^* astrocyte cultures were incubated in glutamate-free, Ca^2+^-containing HBSS medium. After 4 h, the culture supernatant was collected, and glutamate release in the medium was measured (*n* = 7), ** *p* < 0.01 as determined by Mann–Whitney test. (**B**) For evaluation of glutamate uptake, astrocyte cultures were incubated with 100 or 200 µM glutamate in the Ca^2+^-containing HBSS medium (*n* = 5), * *p* < 0.05 as determined by Tukey’s test, results are presented as mean ± SEM.

**Figure 2:**
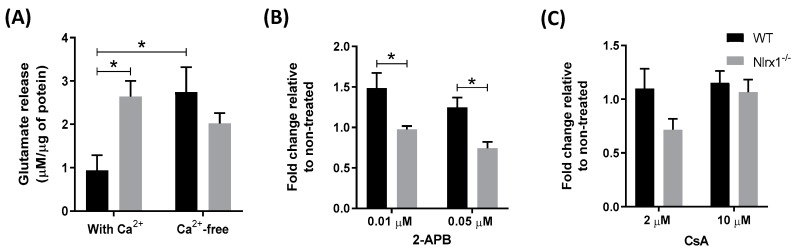
Excess Ca^2+^ release from the ER mediates glutamate release from *Nlrx1^-/-^* astrocytes. (**A**) WT and *Nlrx1^-/-^* astrocytes were incubated in a Ca^2+^-containing HBSS buffer or Ca^2+^-free Lock’s solution for 1 h (*n* = 7); (**B**) astrocyte cultures were incubated with 0.01 or 0.05 µM of 2-APB (*n* = 5), or (**C**) with 2 or 10 µM of CsA (*n* = 6) in the Ca^2+^-free Lock’s solution for 1 h. The supernatant was collected from all cultures and glutamate in the medium was measured by the glutamate assay kit. * *p* < 0.05 as determined by Tukey’s test, results are presented as mean ± SEM.

To determine the role of NLRX1 in the glutamate release and uptake by astrocytes, we incubated primary astrocyte cultures from WT and *Nlrx1^-/-^* mice with or without glutamate in a Ca^2+^-containing medium. Our data shows that in the cultures incubated with the glutamate-free medium for 4 h (Figure 1A) or 1 h (Figure 2A), *Nlrx1^-/-^* astrocytes released significantly higher levels of glutamate compared to WT astrocytes. In the cultures incubated with 100 µM glutamate, there was no significant difference in the glutamate uptake between WT and *Nlrx1^-/-^* astrocytes (Figure 1B). However, when we challenged both cultures with a higher concentration of glutamate (200 µM), WT astrocytes significantly enhanced their glutamate uptake, while there was no significant change in the *Nlrx1^-/-^* astrocytes’ glutamate uptake (Figure 1B). Therefore, after incubation with 200 µM glutamate, WT astrocytes had a significantly higher glutamate uptake (fivefold) than *Nlrx1^-/-^* astrocytes (Figure 1B).

### 3.2. Nlrx1^-/-^ Astrocytes’ Excess Glutamate Release Is Ca^2+^-Dependent

Given that glutamate release by astrocytes is mediated primarily by the elevation of intracellular Ca^2+^ levels [18], first, we examined whether the presence of extracellular Ca^2+^ plays a role in glutamate release from *Nlrx1^-/-^* astrocytes. We incubated WT and *Nlrx1^-/-^* astrocyte cultures in a Ca^2+^-containing or Ca^2+^-free medium for 1 h, followed by the measurement of glutamate in the medium. We observed that removal of Ca^2+^ from the medium resulted in a significant increase in glutamate release from WT astrocytes, while no significant changes in glutamate release were observed in the *Nlrx1^-/-^* astrocytes (Figure 2A). This suggests that the presence of extracellular Ca^2+^ does not have a significant effect on glutamate release in *Nlrx1^-/-^* cultures.

We further assessed whether this glutamate release is mediated by Ca^2+^ release from the intracellular Ca^2+^ stores, including ER and mitochondria. We incubated WT and *Nlrx1^-/-^* astrocyte cultures with different concentrations of 2-Aminoethyl diphenylborinate (2-APB, an inhibitor of inositol-1,4,5-trisphosphate (IP3) receptors that inhibits Ca^2+^ release from the ER) [64] or Cyclosporin A (CsA, an inhibitor of mitochondrial Ca^2+^ release) [65] in the Ca^2+^-free medium. A significant reduction was detected in the glutamate release from 2-APB-treated *Nlrx1^-/-^* astrocytes at both concentrations, compared to WT (Figure 2B). By contrast, in cultures treated with CsA, no significant change was detected in both genotypes (Figure 2C).

### 3.3. Glutamate Release by Nlrx1^-/-^ Astrocytes Is Mediated By Exocytosis

**Figure 3:**
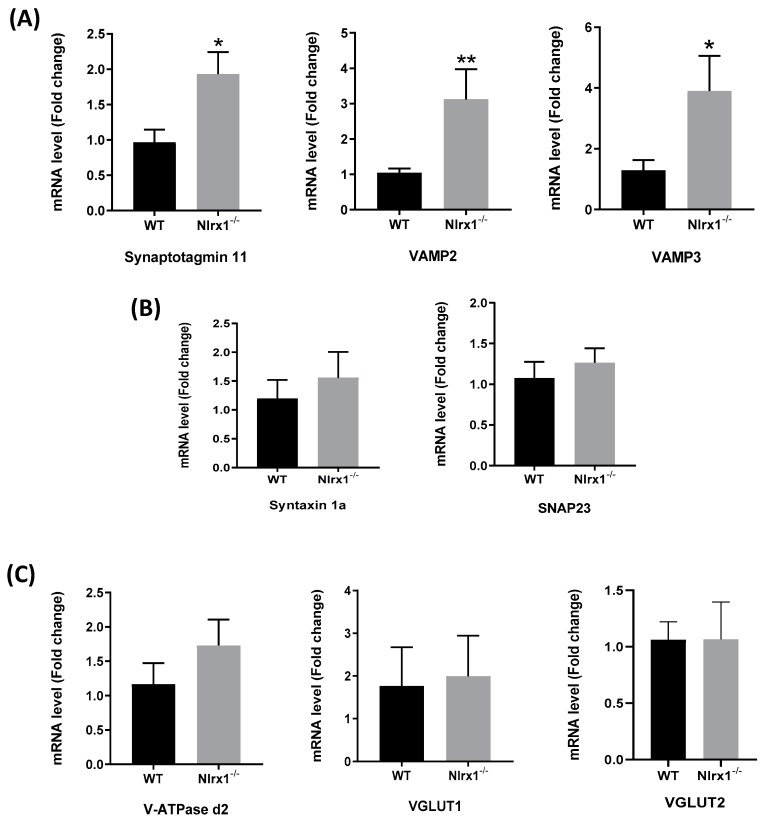
Increased mRNA expression of the proteins of exocytosis in *Nlrx1^-/-^* astrocytes. mRNA expression of the proteins of exocytosis in WT and *Nlrx1^-/-^* astrocytes shows (**A**) significant upregulation of mRNA expression of the Ca^2+^ sensor, synaptotagmin 11, and vesicular fusion proteins, VAMP2 and VAMP3, in *Nlrx1^-/-^* astrocytes compared to WT; (**B**) no significant change in the cell membrane fusion proteins’ mRNA expression; and (**C**) no significant change in the expression of the proteins upstream of Ca^2+^ release from the ER. ** *p* < 0.01 and * *p* < 0.05 as determined by Mann–Whitney test (n ≥ 5), results are presented as mean ± SEM.

Since we found that the NLRX1-mediated glutamate release is Ca^2+^-dependent, we further evaluated whether it is mediated by exocytosis. We measured gene expression of the proteins involved in exocytosis, upstream and downstream of the Ca^2+^ release from the ER, in WT and *Nlrx1^-/-^* astrocyte cultures. The results demonstrated that the mRNA expression of the astrocytic Ca^2+^ sensor, synaptotagmin 11, and the vesicular fusion proteins (VAMP2 and VAMP3) was significantly upregulated in *Nlrx1^-/-^* astrocytes relative to WT (Figure 3A), while no significant change was observed in the mRNA expression of the cell membrane fusion proteins (Syntaxin 1a and SNAP23) (Figure 3B) or the proteins upstream of the Ca^2+^ release (V-ATPase d2, VGLUT1, and VGLUT2) (Figure 3C).

### 3.4. mRNA and Protein Expression of Glutamate Transporters in Astrocytes

**Figure 4:**
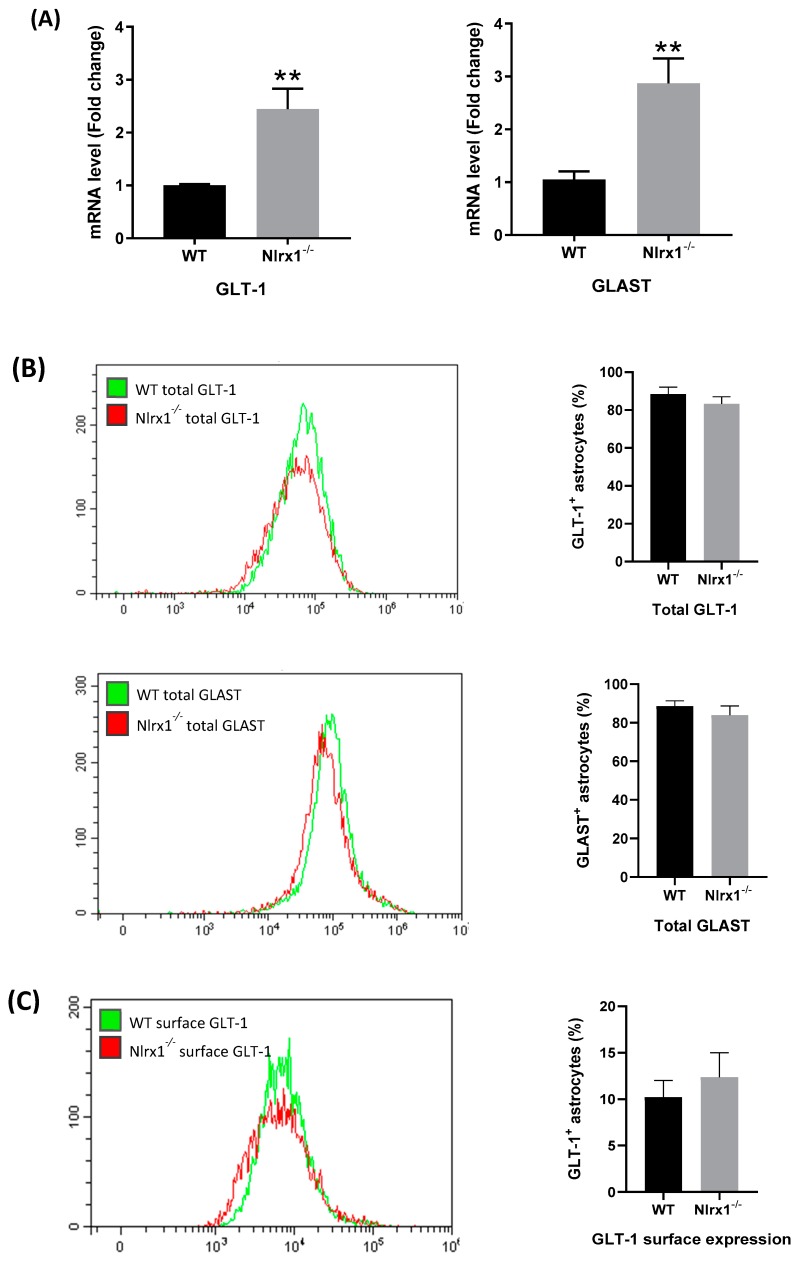

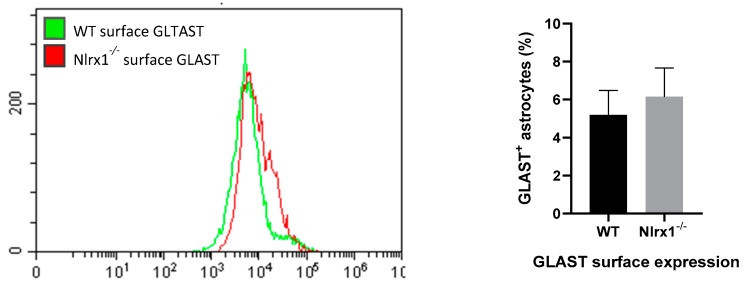
mRNA and protein expression of GLT-1 and GLAST in astrocytes. (**A**) mRNA expression of GLT-1 and GLAST is significantly upregulated in *Nlrx1^-/-^* astrocytes compared to WT (*n* = 5). ** *p* < 0.01 as determined by Mann–Whitney test; (**B**) the total protein expression of GLT-1 and GLAST proteins in WT and *Nlrx1^-/-^* astrocytes was measured by flow cytometry (*n* = 5); (**C**) the cell surface expression of both transporters on astrocytes was measured by flow cytometry (*n* = 7). Representative flow cytometric histograms presented on the left side, *p* > 0.05 as determined by Mann–Whitney test, results are presented as mean ± SEM.

To further investigate the mechanism by which NLRX1 enhances glutamate uptake, we measured the relative gene expression of the astrocytes’ glutamate transporters, GLT-1 and GLAST, in WT and *Nlrx1^-/-^* astrocyte cultures, using qPCR. The mRNA expression of both transporters was significantly higher in *Nlrx1^-/-^* astrocyte cultures relative to WT (Figure 4A). In parallel, we stained WT and *Nlrx1^-/-^* astrocytes with anti-GLT-1 or anti-GLAST antibodies and quantified the total protein expression and the cell surface expression of both transporters by flow cytometry. As shown in Figure 4B,C, no significant change was detected in either GLT-1 or GLAST total protein expression (Figure 4B) or cell surface expression (Figure 4C) between astrocytes of both genotypes.

### 3.5. NLRX1 Enhances Mitochondrial Functions in Astrocytes

**Figure 5:**
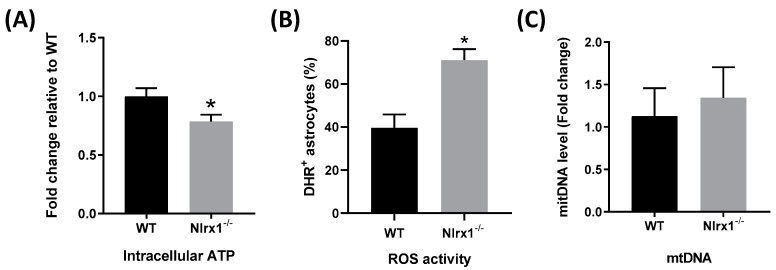
NLRX1 enhances mitochondrial functions in astrocytes. (**A**) The level of intracellular ATP was measured in WT and *Nlrx1^-/-^* astrocytes using an ATP bioluminescent assay kit (*n* = 5); (**B**) the level of oxidative activity was measured in WT and *Nlrx1^-/-^* astrocytes by flow cytometry (*n* = 4); (**C**) the difference between the amount of mtDNA in WT and *Nlrx1^-/-^* astrocytes was measured by qPCR (*n* = 3). * *p* < 0.05 as determined by Mann–Whitney test, results are presented as mean ± SEM.

The optimal function of glutamate transporters requires an enormous amount of energy [12,13]. Accordingly, we measured levels of intracellular ATP in both WT and *Nlrx1^-/-^* astrocytes. We found that *Nlrx1^-/-^* astrocytes have significantly less (20%) intracellular ATP compared to WT (Figure 5A). Since oxidative stress exerts a negative effect on the functional activity of the transporters [66,67], we measured ROS activity in both WT and *Nlrx1^-/-^* astrocytes by flow cytometry. Our results demonstrated that *Nlrx1^-/-^* astrocytes have significantly higher oxidative activity than WT (Figure 5B). Since mitochondria are the major source of intracellular ATP and ROS in the cells, we evaluated the number of mitochondria in WT and *Nlrx1^-/-^* astrocytes. No significant difference was detected in the amount of mtDNA between WT and *Nlrx1^-/-^* astrocytes (Figure 5C).

## 4. Discussion

In this study, we report that the anti-inflammatory [40,41,42,43,44,45,47,48] and prosurvival molecule [49], NLRX1, helps maintain glutamate homeostasis in the CNS. Our findings suggest that NLRX1 enhances astroglial glutamate uptake by promoting the functional activity of glutamate transporters, and inhibits glutamate release from astrocytes by suppressing Ca^2+^-mediated glutamate exocytosis. To our knowledge, this is the first time that one protein has been shown to be implicated in both processes that regulate glutamate homeostasis.

Given that GLT-1 and GLAST are responsible for the uptake of more than 90% of the extracellular glutamate in the CNS [68,69], we measured the mRNA and protein expression of these glutamate transporters in astrocytes from WT and *Nlrx1^-/-^* mice. Unexpectedly, the mRNA expression of both transporters was significantly higher in *Nlrx1^-/-^* astrocytes than WT. However, when we measured the total protein expression, as well as the cell surface expression of both transporters, we did not see any significant difference between astrocytes of both genotypes. These findings suggest that the enhanced glutamate uptake in WT astrocytes cannot be attributed to changes in the transcription or translation of the glutamate transporters. In line with our findings, Conrad and Stoffel reported that the direct phosphorylation of GLAST protein by protein kinase C (PKC) reduces its glutamate uptake activity, while immunofluorescence does not show any effect on its protein expression [70]. Another study revealed that arachidonic acid (AA) downregulates glutamate uptake by EAAT-1 by decreasing its affinity to glutamate and the maximal transport rate approximately 30% with no effect on the expression of its protein [71]. In a third study, Trotti et al. reported that oxidative stress by H_2_O_2_ induces direct oxidation of the sulfhydryl (SH) group of both transporters, which decreases their glutamate uptake with no protein degradation or reduction in their surface expression [72].

Since NLRX1 is localized in the mitochondria, we hypothesized that NLRX1 enhances the glutamate uptake activity of both GLT-1 and GLAST by improving mitochondrial functions in astrocytes. The level of intracellular ATP in astrocytes is one of the crucial factors that determine the functional activity of glutamate transporters [12,13]. Many previous studies reported that ATP depletion, as in cases of brain ischemia, induces glutamate uptake failure caused by loss or reversal of the transporters’ function [73,74]. To pay for its own energy consumption, a large portion of glutamate metabolites in astrocytes is consumed in the tricarboxylic acid (TCA) cycle to produce more ATP [75]. Interestingly, we found that *Nlrx1^-/-^* astrocytes contain 20% less ATP that WT astrocytes, which could either be a cause and/or a result of the reduced glutamate uptake by these cells. Nevertheless, these data, in agreement with the previous studies [50,51], suggest that NLRX1 enhances mitochondrial ATP production.

Moreover, it was reported that the intracellular oxidative stress induced by H_2_O_2_ reduces the functional activity of glutamate transporters [66,67]. In this regard, we measured the level of ROS activity in both WT and *Nlrx1^-/-^* astrocytes. Our results demonstrated that *Nlrx1^-/-^* astrocytes have 50% more oxidative activity than WT, which could be responsible for the significant deficiency of their glutamate uptake. Furthermore, since there was no difference in the level of mtDNA between WT and *Nlrx1^-/-^* astrocytes, we excluded the possibility that NLRX1 increases the number of mitochondria.

Taken together, our results suggest that NLRX1 enhances mitochondrial functions in astrocytes, and thus boosts the functional activity of both GLT-1 and GLAST, rather than their protein expression. This effect of NLRX1 on the transporters is achieved by suppressing oxidative stress and, partially, by maintaining sufficient ATP production.

Recent studies suggest that astrocytes express components necessary for the Ca^2+^-mediated exocytosis, which is the principal mechanism of astroglial glutamate release under physiological conditions [4,17,18,19]. We evaluated whether the excess glutamate release from *Nlrx1^-/-^* astrocytes is Ca^2+^-dependent. First, to exclude the role of extracellular Ca^2+^, we incubated astrocytes in a Ca^2+^-free instead of the Ca^2+^-containing medium. We observed that WT astrocytes significantly upregulated their glutamate release after Ca^2+^ removal, which agrees with the previously published report by Kostic et al. (2017) [76]. The removal of Ca^2+^ from the medium stimulates Ca^2+^ release from the intracellular Ca^2+^ stores and results in augmentation of glutamate release from astrocytes [76]. However, this does not explain the phenotype in *Nlrx1^-/-^* astrocytes, as there was no change between their glutamate release in the Ca^2+^-containing and the Ca^2+^-free media, probably because they had already reached their maximum capacity of glutamate release, and Ca^2+^ removal does not result in any additional effect. In the second step, we evaluated whether this glutamate release occurs in response to Ca^2+^ release from the intracellular Ca^2+^ stores. We found that inhibiting Ca^2+^ release from the mitochondria does not exhibit any significant effect while inhibiting Ca^2+^ release from the ER reduces the excess glutamate release from *Nlrx1^-/-^* astrocytes. These observations corroborate previous findings that ER Ca^2+^ plays an essential role in glutamate release [4,17].

Consequently, *Nlrx1^-/-^* astrocytes show higher mRNA expression of exocytosis proteins downstream of Ca^2+^ release from the ER, including the Ca^2+^ sensor, synaptotagmin 11, and the vesicular fusion proteins (VAMP2 and VAMP3). In contrast, there is no significant change in the expression of the cell membrane fusion proteins (Syntaxin 1 a and SNAP23), or the proteins preceding Ca^2+^ release from the ER (V-ATPase d2, VGLUT1, and VGLUT2). Therefore, our results suggest that the excess glutamate release from *Nlrx1^-/-^* astrocytes is mediated by excess Ca^2+^ release from the ER, followed by an augmentation in the expression of the molecules of exocytosis as a result of the excess Ca^2+^ release. Collectively, these data provide evidence that NLRX1 mediates its inhibitory effect on glutamate release from astrocytes mainly by suppressing Ca^2+^ release from the ER, which consequently suppresses glutamate exocytosis.

The connection between the two mechanisms by which NLRX1 mediates its effects on astroglial glutamate uptake and release is still unclear. Being situated in the mitochondria, it is plausible that NLRX1 modifies mitochondrial functions [44,49,50,51]. However, the mechanism by which NLRX1 modifies the function of the ER requires more in-depth investigations.

**Figure 6:**
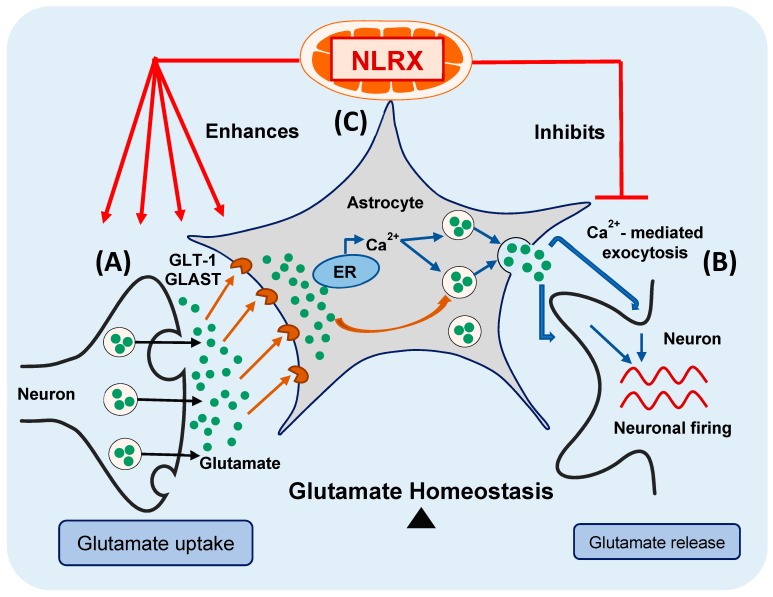
NLRX1 maintains glutamate homeostasis in the CNS. (**A**) Uptake of the extracellular glutamate by astrocytes is mediated by the glutamate uptake transporters (GLT-1 and GLAST); (**B**) Ca^2+^-mediated exocytosis mediates glutamate release from astrocytes, in response to Ca^2+^ release from the ER. The released glutamate helps to synchronize and reinforce the firing of the surrounding neurons; (**C**) NLRX1 enhances astroglial glutamate uptake and inhibits its Ca^2+^-mediated glutamate exocytosis, hence maintaining glutamate homeostasis in the CNS.

In conclusion, in the current study, we provide evidence that NLRX1 enhances astroglial glutamate uptake and inhibits excess glutamate release from astrocytes, thus maintaining glutamate homeostasis in the CNS (Figure 6—modified from the graphical abstract of our recent publication) [4]. Consequently, NLRX1 represents a potential therapeutic target for the inflammatory and neurodegenerative diseases associated with glutamate excitotoxicity in the CNS.

## Figures and Tables

**Table 1:** Primer sequences used for qPCR.

*18S*	F: 5′ CGG CTA CCA CAT CCA AGG AA ′3 R: 5′ GCT GGA ATT ACC GCG GCT ′3
Exocytosis	
*V-ATPase d2*	F: 5′ TTC AGT TGC TAT CCA GGA CTC GGA ′3 R: 5′ GCA TGT CAT GTA GGT GAG AAA TGT GCT CA ′3
*VGLUT1*	F: 5′ GGT GGA GGG GGT CAC ATA C ′3 R: 5′ AGA TCC CGA AGC TGC CAT AGA ′3
*VGLUT2*	F: 5′ CCC TGG AGG TGC CTG AGA A ′3 R: 5′ GCG GTG GAT AGT GCT GTT GTT ′3
*Synaptotagmin 11*	F: 5′ GAC ACT TGC CGA AGA TGG ATA TC ′3 R: 5′ TGC GTT TTC TGC CGT AGT AGA ′3
*VAMP2*	F: 5′ CAC AAT CTG GTT CTT TGA GGA G ′3 R: 5′ AGA GAC TTC AGG CAG GAA TTA G ′3
*VAMP3*	F: 5′ CTC ACC AAG GCA TCA GTC TG ′3 R: 5′ ATT CTA AGA GCA CCA GGC ATC ′3
*Syntaxin 1a*	F: 5′ TCC AAG CTA AAG AGC ATT GAG C ′3 R: 5′ GGC GTT GTA CTC GGA CAT GA ′3
*SNAP23*	F: 5′ AAT CCT GGG TTT AGC CAT TGA GTC ′3 R: 5′ TTG GTC CAT GCC TTC TTC TAT GC ′3
Glutamate transporters	
*GLT-1*	F: 5′ CGA TGA GCC AAA GCA CCG AA ′3 R: 5′ CTG GAG ATG ATA AGA GGG AGG ATG ′3
*GLAST*	F: 5′ TCA AGT TCT GCC ACC CTA CC ′3 R: 5′ TCT GTC CAA AGT TCA GGT CAA ′3

## Abbreviations

CNS	Central nervous system
EAAT1 and EAAT2	Excitatory amino acid transporters 1 and 2
GLAST	Glutamate–aspartate transporter
GLT-1	Glutamate transporter-1
ATP	Adenosine triphosphate
VGLUT1 and VGLUT2	Vesicular glutamate transporters 1 and 2
V-ATPase	Vacuolar (H+) ATPase
VAMP2 and VAMP3	Vesicle-associated membrane protein 2 and 3
ER	Endoplasmic reticulum
SNAP23	Soluble N-ethylmaleimide-sensitive factor attachment protein 23
HIV	Human immunodeficiency virus
MS	Multiple sclerosis
ALS	Amyotrophic lateral sclerosis
AD	Alzheimer’s disease
PD	Parkinson’s disease
NLRs	NOD-like receptors
NF-κB	Nuclear factor-κB
NLRX1	NOD-like receptor X1
IBD	Inflammatory bowel disease
EAE	Experimental autoimmune encephalomyelitis
WT	Wild-type
*Nlrx1^-/-^*	Nlrx1 knockout
dFBS	Deactivated fetal bovine serum
DMSO	Dimethyl sulfoxide
HBSS	Hank’s Balanced Salt Solution
qPCR	Quantitative real-time PCR
PBS	Phosphate-buffered saline
ROS	Reactive oxygen species
DHR	Dihydrorhodamine 123
mtDNA	Mitochondrial DNA
SEM	Standard error of the mean
2-APB	2-Aminoethyl diphenylborinate
IP3	Inositol-1,4,5-trisphosphate
CsA	Cyclosporin A
PKC	Protein kinase C
AA	Arachidonic acid
SH group	Sulfhydryl group
TCA	Tricarboxylic acid
